# Transverse Mode Instability in High-Power Yb-Doped Double-Clad Fiber Amplifiers: A Three-Layer Optical–Thermal Analysis Based on Stimulated Thermal Rayleigh Scattering †

**DOI:** 10.3390/mi17030326

**Published:** 2026-03-05

**Authors:** Elbis Santos Cardoso, Ricardo Elgul Samad, Cláudio Costa Motta

**Affiliations:** 1 LaMP—Power Microwave and Photonics Laboratory, University of São Paulo (USP), São Paulo 05508-000, Brazil; ccmotta@usp.br; 2 Center for Lasers and Applications (CLA), Nuclear and Energy Research Institute (IPEN–CNEN), São Paulo 05508-000, Brazil; resamad@ipen.br

**Keywords:** stimulated thermal rayleigh scattering, transverse mode instability, double-clad fibers, quantum defect heating, LP modes

## Abstract

Transverse mode instability (TMI) in high-power ytterbium-doped double-clad fiber lasers is widely interpreted as being a consequence of a thermo-optic nonlinear phenomenon driven by stimulated thermal Rayleigh scattering. This work presents a coupled optical–thermal model for a continuous-wave forward-pumped ($\lambda_{p}=976\;\mathrm{nm}$) fiber amplifier emitting at $\lambda_{s}=1064\;\mathrm{nm}$ over an optimal length of 12 m. The formulation explicitly resolves the three radial regions of a double-clad fiber, avoiding single-clad approximations. Modal fields are computed using the weakly guiding approximation (WGA) in the core combined with the semi-WGA at the cladding interfaces, enabling accurate calculation of higher-order modes of penetration into the inner cladding and of the transverse eigenvalues $U_{01}$ and $U_{mn}$ relevant to TMI. Within this framework, the nonlinear stimulated thermal Rayleigh scattering coupling coefficient is evaluated, including gain saturation and the thermal eigenmodes of the multi-layer geometry. The results show that the inner cladding modifies both the optical and thermal mode structures, altering the optical–thermal overlap between ${\mathrm{LP}}_{01}$ and higher-order modes and changing the effective strength of STRS, directly influencing the predicted TMI threshold. The proposed formulation provides a quantitative and physically consistent tool for analyzing thermo–optic dynamics in Yb-double-clad fiber amplifiers and supports the design of next-generation high-power fiber lasers with improved modal stability.

## 1. Introduction

High-power ytterbium-doped double-clad fiber lasers (YDCFLs) have enabled continuous-wave operation well above the kilowatt level and are widely employed in industrial and scientific applications [1]. This architecture is recognized for its high efficiency, robustness, and power scalability, and it has become the leading choice for high-brightness systems [2,3,4,5,6]. Despite these advantages, YDCFLs are ultimately limited by thermally driven effects, among which transverse mode instability (TMI) remains the primary obstacle to further power scaling in large-mode-area (LMA) fibers. TMI manifests as an abrupt transfer of energy from the fundamental mode ${\mathrm{LP}}_{01}$ (the linearly polarized mode of radial order zero and azimuthal order one) to higher-order modes (HOMs), typically accompanied by kilohertz-scale oscillations in modal content [7,8,9,10,11].

A broad consensus that TMI is driven by the nonlinear mechanism of stimulated thermal Rayleigh scattering (STRS) has emerged in the literature. In this process, interference between guided modes generates a longitudinally modulated irradiance pattern, which, through quantum-defect heating, induces a dynamic refractive-index perturbation in the doped core [7,12,13,14,15,16]. Radial heat diffusion introduces a phase delay between the irradiance modulation and the corresponding refractive-index response, forming a traveling thermo-optic grating that enables coherent modal coupling and produces the characteristic threshold behavior of TMI. Recent refinements have incorporated gain saturation, improved thermal-source descriptions, and rigorous treatments of radial thermal modes [17].

In a substantial proportion of prior work, researchers modeled double-clad fibers (DCF) using an effective two-region approximation, an approach equivalent to treating the waveguide as a single-clad fiber (SCF) comprising only a core and a single cladding [13,18,19,20,21,22,23]. This approach has been instrumental in elucidating many aspects of STRS and TMI. However, it cannot fully reveal the role of the inner cladding, which modifies the transverse eigenvalue structure and may allow HOMs to penetrate beyond the core under high-power operation [11,24,25]. These effects alter optical overlap integrals and influence susceptibility to STRS-driven coupling.

More advanced modal descriptions rely on the weakly guiding approximation (WGA) within the core region, combined with semi-weakly guiding approximation (semi-WGA) boundary conditions to account for the refractive-index contrast at the core and inner- and outer-cladding interfaces [24,26,27,28]. These multi-layer formulations yield accurate propagation constants and field distributions, enabling realistic computation of optical–thermal overlap integrals and STRS coupling coefficients in double-clad fibers.

In this extended version of our previous conference work [29], we report the development of a quantitative model that describes the influence of STRS on TMI in double-clad fibers. This model combines WGA-based core solutions with semi-WGA boundary conditions at both cladding interfaces, together with thermal diffusion induced by the quantum defect and the STRS formalism governing coupling between ${\mathrm{LP}}_{01}$ and HOMs. This framework enables a physically consistent evaluation of how the multi-layer geometry modifies the optical–thermal modal structure and influences the TMI threshold. We then apply the model to analyze a high-power forward-pumped fiber amplifier operating at $\lambda_{p}=976\;\mathrm{nm}$ and emitting at $\lambda_{s}=1064\;\mathrm{nm}$ in a continuous-wave (CW) steady-state regime over an active fiber length of 12 m. The system is based on a large-mode-area ytterbium-doped double-clad fiber (Yb-DCF), with a radial geometry defined by a core radius a=12.5μm, an inner cladding radius b=125μm, and an outer cladding radius c=200μm. The insights provided in this analysis can aid in the design of next-generation high-power fiber lasers with improved modal stability.

## 2. Theoretical Model

**Figure 1:**
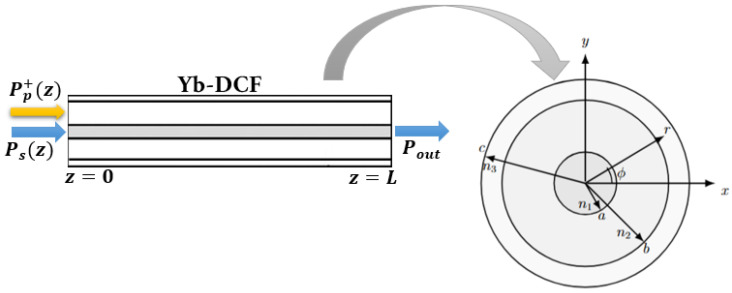
Schematic representation of a high-power Yb-doped double-clad fiber amplifier, with core (0<r<a), inner cladding (a<r<b), and outer cladding (b<r<c). Forward pumping is applied, with the pump power $P_{p}^{+}\left(z\right)$ injected at z=0 into the inner cladding and progressively absorbed along the active fiber. A seed signal $P_{s}\left(z\right)$ is injected in the forward direction at z=0, and it is amplified toward z=L, where the output signal power $P_{\mathrm{out}}$ is extracted. On the right, the fiber cross-section is shown, indicating the radii *a*, *b*, and *c* and the refractive indices $n_{1}$, $n_{2}$, and $n_{3}$ defining the multi-layer radial geometry.

Describing transverse mode instability in ytterbium-doped fiber amplifiers requires joint modeling of the guided optical fields, heat generation and diffusion in the core, and the thermo-optic coupling between modes induced by STRS. We explicitly consider a three-layer fiber—a core, inner cladding, and outer cladding—with refractive indices $n_{1}>n_{2}>n_{3}$, respectively, a characteristic structure of double-clad fibers employed in high-power systems. This geometry, illustrated in Figure 1, captures the role of the inner cladding in modifying modal confinement and the thermal eigenmodes relevant to STRS while retaining the nearly uniform thermal diffusion properties of the silica regions. Although the thermal conductivities of the core and inner cladding are nearly identical, the presence of the intermediate layer plays an important role in the harmonic thermal response relevant to STRS. The additional boundary condition at r=b modifies the thermal eigenvalues $\eta_{ml}$ and therefore the phase-lagged temperature component that drives thermo-optic coupling. Thus, even though steady-state heat diffusion remains almost uniform in the glass regions, the multi-layer geometry has a significant impact on the dynamic thermal modes involved in TMI.

### 2.1. Modal Formulation in Three-Layer Optical Fibers

The modal analysis employed in this work follows the vector formulation reported by Tsao et al. [27], which describes electromagnetic propagation through the Debye potential and rigorously enforces the boundary conditions of the tangential components of the electric and magnetic fields at the interfaces r=a and r=b. This approach remains valid even when the refractive-index contrast between layers is high, a typical condition in double-clad fibers.

From Maxwell’s equations and the separation of variables in cylindrical coordinates, the normalized parameters can be obtained [24,26,27]:

(1) $$U_{1}^{2}=a^{2}\left(k_{0}^{2}n_{1}^{2}-\beta^{2}\right),\;U_{2}^{2}=a^{2}\left(k_{0}^{2}n_{2}^{2}-\beta^{2}\right),\;W_{3}^{2}=b^{2}\left(\beta^{2}-k_{0}^{2}n_{3}^{2}\right),$$

where $k_{0}=2\pi /\lambda$ is the wavenumber in a vacuum. The parameters $U_{1}$, $U_{2}$, and $W_{3}$ characterize propagation in the core, penetration into the inner cladding, and evanescent decay in the outer cladding, respectively.

The propagation constant β is obtained by numerically solving the characteristic (eigenvalue) equations arising from the enforcement of the electromagnetic boundary conditions at r=a and r=b. Once found, the resulting profiles may be treated in the LP form, provided that the weak-guidance approximation is applied only at the core–inner-cladding interface, where $n_{1}\approx n_{2}$. At r=b, the larger index contrast between $n_{2}$ and $n_{3}$ requires the use of the full boundary conditions of the vector model. This combination enables an accurate description of modal behavior throughout the structure, which is essential for evaluating the optical and thermal overlap terms involved in STRS.

These modal solutions also determine the transverse eigenvalues $U_{01}$ and $U_{mn}$, which are entered into the asymptotic TMI-threshold model (Equation (31)). Because STRS depends on the spatial interference between modes and on the projection of the thermal source onto the thermal eigenmodes, an accurate description of the field penetration into the inner cladding is essential for predicting the strength of thermo-optic coupling.

The resulting fields are then normalized to generate the radial profiles $f_{mn}\left(r\right)$ used in the optical and thermal integrals. The normalization follows the procedure adopted by Dong [13] over the entire cross-section (0<r<c):

(2) $$N_{01}=2\pi \int_{0}^{c}f_{01}^{2}\left(r\right)\;r\;dr,$$

(3) $$N_{mn}=\pi \int_{0}^{c}f_{mn}^{2}\left(r\right)\;r\;dr,\;\left(m>0\right).$$

With this normalization, the scalar field used in the integrals of interference, heat generation, and thermo-optic coupling is expressed as

(4) $$e_{mn}\left(r,\phi \right)=\frac{f_{mn}\left(r\right)\cos\left(m\phi \right)}{\sqrt{2\;n_{\mathrm{eff}}\varepsilon_{0}c\;N_{mn}}}.$$

where *c* is the speed of light in a vacuum, $\varepsilon_{0}$ is the permittivity of free space, and $n_{\mathrm{eff}}$ is the effective refractive index of the LP_*mn*_ mode.

### 2.2. Transverse Fields in the Three-Layer Structure (DCF)

Determining the ${\mathrm{LP}}_{mn}$ modes requires computing the normalized parameters $U_{1}$, $U_{2}$, and $W_{3}$ obtained from the same characteristic (dispersion) equations that ensure continuity of the tangential components of the electric and magnetic fields at the interfaces r=a and r=b.

At r=a, due to the small index contrast $n_{1}\approx n_{2}$, the weak-guidance approximation remains valid. At the interface r=b, however, the contrast $n_{2}>n_{3}$ is significant, making the WGA inadequate and justifying the use of the semi-weak guidance approximation, as described by Tan et al. [24]:

(5) $$E_{y1}\left(r,\phi \right)=A\;J_{m}\;\left(U_{1}\frac{r}{a}\right)\cos\left(m\phi \right),\;\mathrm{for}\;0<r\leq a\;\left(\mathrm{core}\right)$$

(6) $$E_{y2}\left(r,\phi \right)=\left[B_{1}\;J_{m}\;\left(U_{2}\frac{r}{a}\right)+B_{2}\;N_{m}\;\left(U_{2}\frac{r}{a}\right)\right]\cos\left(m\phi \right),\;\mathrm{for}\;a<r\leq b\;(\mathrm{inner}\;\mathrm{cladding})$$

(7) $$E_{y3}\left(r,\phi \right)=C\;K_{m}\;\left(W_{3}\frac{r}{b}\right)\cos\left(m\phi \right),\;\mathrm{for}\;b<r<c\;(\mathrm{outer}\;\mathrm{cladding})$$

where $J_{m}$, $N_{m}$, and $K_{m}$ denote the Bessel, Neumann, and modified Bessel functions, respectively. The coefficients *A*, $B_{1}$, $B_{2}$, and *C* are determined by enforcing continuity of the tangential components of the electromagnetic fields at the interfaces r=a and r=b, resulting in four coupled equations. Although the thermal conductivity of the silica layers is nearly identical, the presence of the inner cladding introduces an additional thermal interface in the harmonic heat equation. This modifies the thermal eigenvalues $\eta_{ml}$ associated with STRS and slightly changes the radial shape of the thermal modes. As a result, the effective phase delay between irradiance modulation and temperature response differs from that of a two-layer SCF, contributing to the nonlinear coupling coefficient $\chi_{mn}$, even though the steady-state heat flow is nearly uniform within the silica regions.

One of the characteristic (dispersion) equations applied at the interface r=b can be expressed as follows [24]:

(8) $$\begin{array}{cc} \; & \frac{A\;U_{1}}{U_{2}J_{m}\left(U_{1}\right)}\left[J_{m+1}\left(U_{1}\right)\cos\left[\left(m+1\right)\phi \right]-J_{m-1}\left(U_{1}\right)\cos\left[\left(m-1\right)\phi \right]\right]\\ \; & \;\;=A_{21}\left[J_{m+1}\left(U_{2}\right)\cos\left[\left(m+1\right)\phi \right]-J_{m-1}\left(U_{2}\right)\cos\left[\left(m-1\right)\phi \right]\right]\\ \; & \;\;\;+A_{22}\left[N_{m+1}\left(U_{2}\right)\cos\left[\left(m+1\right)\phi \right]-N_{m-1}\left(U_{2}\right)\cos\left[\left(m-1\right)\phi \right]\right], \end{array}$$

This represents one of the continuity conditions that define the characteristic equation of the mode in the three-layer structure.

The presence of the inner cladding allows greater radial penetration of the fields and redistributes the optical power between the core and the inner cladding. This modifies the spatial overlap between ${\mathrm{LP}}_{01}$ and higher-order modes, as well as the overlap with the thermally induced index perturbation and the gain profile. As a consequence, the real part of the nonlinear coupling coefficient $\chi_{mn}$ and the associated TMI threshold become functions of the full three-layer optical–thermal structure.

### 2.3. Modal Superposition and Interference Pattern

Considering the normalized modal fields $e_{mn}\left(r,\phi \right)$, the total optical field within the core region (0≤r≤a) can be written as [13]

(9) $$E\left(r,\phi ,z,t\right)=\sum_{m,n}\sqrt{P_{mn}\left(z\right)}\;e_{mn}\left(r,\phi \right)\;e^{i(\beta_{mn}z-\omega_{mn}t)}+c.c.,$$

where $P_{mn}\left(z\right)$ is the power carried by the ${\mathrm{LP}}_{mn}$ mode, $\omega_{mn}$ is its optical frequency, and $\beta_{mn}$ is the corresponding propagation constant.

The interference between the fundamental mode ${\mathrm{LP}}_{01}$ and the ${\mathrm{LP}}_{mn}$ modes yields the irradiance pattern

(10) $$\begin{array}{cc} I(r,\phi ,z,t)\approx \; & \sum_{m=0}^{\infty }\sum_{n=1}^{\infty }\frac{P_{mn}\left(z\right)}{N_{mn}}\;f_{mn}^{2}\left(r\right)\;\cos^{2}\left(m\phi \right)\\ \; & +2\sum_{m=1}^{\infty }\sum_{n=1}^{\infty }\sqrt{P_{01}\left(z\right)\;P_{mn}\left(z\right)}\;\frac{f_{01}\left(r\right)\;f_{mn}\left(r\right)}{\sqrt{N_{01}N_{mn}}}\cos\left(m\phi \right)\\ \; & \;\times \cos\;\left[\left(\beta_{mn}-\beta_{01}\right)z-\left(\omega_{mn}-\omega_{01}\right)t\right], \end{array}$$

where

(11) $$q=\beta_{mn}-\beta_{01},\;\Omega =\omega_{mn}-\omega_{01}.$$

The term cos(qz−Ωt) represents a “traveling irradiance grating” arising from modal beating. Its spatial period is determined by the modal beat length:

(12) $$\Lambda_{01,mn}=\frac{2\pi }{\left|q\right|}.$$

This space–time modulation of the optical irradiance is the fundamental driver of STRS [7,13]. Through quantum-defect heating, irradiance beating produces a spatially and temporally oscillating thermal source. The harmonic thermal response of the medium then generates a delayed temperature modulation, which induces a refractive-index perturbation capable of coherently coupling the optical modes. Thus, Equation (10) constitutes the starting point for the thermo-optic dynamics leading to TMI.

#### Smith & Smith’s Moving-Grating Hypothesis

Smith & Smith [7] demonstrated that a phase shift between the irradiance pattern and the induced thermo-optic grating is a necessary condition for coherent power transfer between modes. This phase shift arises whenever the irradiance modulation travels along the fiber due to a slight difference in optical frequency between the interfering modes.

By identifying that

(13) $$\omega_{1}\equiv \omega_{01},\;\omega_{2}\equiv \omega_{mn},$$

we can express the axial velocity of the moving irradiance grating as follows [7]:

(14) $$v_{\mathrm{grating}}=\frac{c}{\omega_{01}}\frac{\omega_{mn}-\omega_{01}}{n_{\mathrm{eff},mn}-n_{\mathrm{eff},01}},$$

where $n_{\mathrm{eff},01}$ and $n_{\mathrm{eff},mn}$ are the effective indices of the ${\mathrm{LP}}_{01}$ and ${\mathrm{LP}}_{mn}$ modes, respectively. For $n_{\mathrm{eff},01}>n_{\mathrm{eff},mn}$, the condition $\omega_{mn}<\omega_{01}$ results in a grating traveling in the positive *z* direction, while $\omega_{mn}>\omega_{01}$ reverses the direction of motion.

In the thermal regime, heat diffusion introduces a characteristic delay in the medium’s thermo-optic response. Consequently, the temperature grating does not perfectly follow the moving optical pattern a phase lag. This broken symmetry leads to a nonreciprocal nonlinear coupling term between the interacting modes. Dong [13] formalized this mechanism by showing that modal interference generates a traveling optical intensity wave of the form cos(qz−Ωt), while the harmonic solution of the heat equation produces a temperature perturbation proportional to $e^{i(qz-\Omega t)}$ with a complex-valued amplitude, whose imaginary part encapsulates the diffusion-induced phase delay.

This phase-lagged thermal response is precisely the origin of the nonlinear coefficient $\chi_{mn}$ responsible for the exponential growth of the higher-order mode above the instability threshold.

### 2.4. Heat Generation and Quantum Defect

The traveling interference pattern discussed in the previous section modulates the local heat deposition in the doped core. The heating rate is determined by the ytterbium ions’ Stokes shift, and the fraction converted into heat is quantified by the quantum defect [30,31]:

(15) $$q_{D}=\left(\frac{\lambda_{s}}{\lambda_{p}}-1\right),$$

which is positive for Yb-doped fiber lasers operating at $\lambda_{s}>\lambda_{p}$.

Interference between ${\mathrm{LP}}_{01}$ and ${\mathrm{LP}}_{mn}$ produces an irradiance modulation with axial periodicity 2π/q and temporal frequency Ω. The harmonic thermal source is written as [13]

(16) $$Q_{T}\left(r,\phi ,z,t\right)=\tilde{Q}\left(r,z\right)\;e^{im\phi }\;e^{i(qz-\Omega t)},$$

with radial amplitude

(17) $$\tilde{Q}\left(r,z\right)=\frac{1}{2}\;q_{D}\;\tilde{g}\left(r,z\right)\;f_{01}\left(r\right)\;f_{mn}\left(r\right)\;\sqrt{\frac{P_{01}\left(z\right)P_{mn}\left(z\right)}{N_{01}N_{mn}}},\;0<r<a,$$

and $\tilde{Q}\left(r,z\right)=0$ for r≥a. Here, $\tilde{g}\left(r,z\right)$ denotes the saturated effective gain that determines the fraction of absorbed pump power contributing to the harmonic component of the heating relevant to STRS, while $f_{01}\left(r\right)$ and $f_{mn}\left(r\right)$ are the normalized modal profiles of the interfering modes.

This oscillatory heating term excites the radial thermal eigenmodes of the fiber, generating a temperature perturbation with the same axial and azimuthal dependencies. The delayed thermo-optic grating arising from this temperature response constitutes the fundamental physical mechanism of STRS-driven modal coupling [7,13,32].

### 2.5. Harmonic Heat Equation and Thermal Modes

The temperature perturbation ΔT generated by $Q_{T}$ obeys the heat equation [7,13,32,33]:

(18) $$\rho C\frac{\partial \Delta T}{\partial t}-\kappa_{0}\nabla_{T}^{2}\Delta T=Q_{T},$$

where ρ is the density, *C* is the specific heat, and $\kappa_{0}$ is the thermal conductivity of the glass. Since $Q_{T}$ contains the factor $e^{im\phi }e^{i(qz-\Omega t)}$, we seek a time-harmonic steady-state solution with the same axial and azimuthal dependence:

(19) $$\Delta T\left(r,\phi ,z,t\right)=e^{im\phi }e^{i(qz-\Omega t)}\sum_{l=1}^{\infty }a_{l}\left(\Omega ,z\right)\;T_{ml}\left(r\right),$$

where $T_{ml}\left(r\right)$ are the radial thermal modes of the three-layer structure.

These modes are obtained from the static heat equation subject to the boundary conditions described below, following the multi-layer thermal formulation widely adopted in high-power double-clad fiber amplifiers [34,35]. At the fiber axis (r=0), cylindrical symmetry is imposed:

(20) $${\left.\frac{\partial T}{\partial r}\right|}_{r=0}=0.$$

At the material interfaces (r=a) and (r=b), continuity of both temperature and heat flux is enforced:

(21) $$T_{i}=T_{i+1},\;k_{i}\frac{\partial T_{i}}{\partial r}=k_{i+1}\frac{\partial T_{i+1}}{\partial r},$$

where the subscript denotes the fiber region (1 for the core, 2 for the inner cladding, and 3 for the outer cladding), and $k_{i}$ is the corresponding thermal conductivity of each region. At the outer-cladding boundary (r=c), heat removal by the environment is modeled through a convective boundary condition,

(22) $$-k_{3}{\left.\frac{\partial T_{3}}{\partial r}\right|}_{r=c}=h\;\left[T_{3}\left(c\right)-T_{c}\right],$$

where *h* is the effective convective heat-transfer coefficient and $T_{c}$ is the coolant temperature.

The corresponding eigenvalues $\eta_{ml}$ incorporate the influence of the inner cladding on radial diffusion and govern the phase-lagged thermal response relevant to STRS.

Projecting the heat equation onto $T_{ml}\left(r\right)$ and using radial orthogonality yield

(23) $$a_{l}\left(\Omega ,z\right)=\frac{\int_{0}^{a}\tilde{Q}\left(r,z\right)\;T_{ml}\left(r\right)\;r\;dr}{\left[\kappa_{0}\left(q^{2}+\eta_{ml}^{2}\right)-i\Omega \rho C\right]\int_{0}^{c}T_{ml}^{2}\left(r\right)\;r\;dr}.$$

It is convenient to introduce the thermal damping parameter,

(24) $$\Gamma_{ml}=\frac{2\kappa_{0}}{\rho C}\left(q^{2}+\eta_{ml}^{2}\right),$$

which makes the frequency dependence explicit in the coupling expressions that enter the nonlinear coefficient $\chi_{mn}$.

### 2.6. Thermo-Optic Perturbation

The temperature variation induces a refractive-index perturbation through the thermo-optic coefficient $k_{T}=\partial n/\partial T$:

(25) $$\Delta n\left(r,\phi ,z,t\right)=k_{T}\;\Delta T\left(r,\phi ,z,t\right).$$

Since ΔT inherits the dependence $e^{im\phi }e^{i(qz-\Omega t)}$, the index perturbation forms a traveling thermo-optic grating that mediates stimulated thermal Rayleigh scattering, providing the link between modal interference and coherent modal coupling.

### 2.7. Nonlinear Modal Coupling Coefficient

The thermo-optic perturbation Δn induces coherent coupling between the ${\mathrm{LP}}_{01}$ and ${\mathrm{LP}}_{mn}$ modes. This interaction is described by the complex nonlinear coefficient $\chi_{mn}\left(z,\Omega \right)$, which, for the three-layer DCF, takes the following form [13,15,31,33,36]:

(26) $$\begin{array}{cc} \chi_{mn}\left(z,\Omega \right)\; & =\frac{k_{0}\;k_{T}}{\pi \kappa_{0}}\left(\frac{\lambda_{s}}{\lambda_{p}}-1\right)\sum_{l=1}^{\infty }F_{ml}\left(\Omega \right)\\ \; & \;\times \frac{\left[\int_{0}^{a}\tilde{g}\left(r,z\right)\;f_{01}\left(r\right)f_{mn}\left(r\right)T_{ml}\left(r\right)\;r\;dr\right]\left[\int_{0}^{c}f_{01}\left(r\right)f_{mn}\left(r\right)T_{ml}\left(r\right)\;r\;dr\right]}{N_{01}N_{mn}\int_{0}^{c}T_{ml}^{2}\left(r\right)\;r\;dr}. \end{array}$$

The complex frequency response is

(27) $$F_{ml}\left(\Omega \right)=\frac{2\left(2\Omega /\Gamma_{ml}-i\right)}{1+{\left(2\Omega /\Gamma_{ml}\right)}^{2}},$$

whose real part peaks at $\Omega ≃\Gamma_{ml}/2$, while the imaginary part captures the diffusion-induced phase delay between the irradiance and thermal gratings. This phase asymmetry underlies the nonreciprocal character of STRS-driven coupling.

### 2.8. Coupled Equations for Power Evolution

In the quasi-stationary regime, the axial evolution of the modal powers is described by the coupled equations [13,17,31,33,37]:

(28) dP01dz=g01(z)P01−g01(z)Reχmn(z,Ω)P01Pmn,

(29) dPmndz=gmn(z)−αmnPmn+g01(z)Reχmn(z,Ω)P01Pmn,

where $g_{01}\left(z\right)$ and $g_{mn}\left(z\right)$ are the saturated modal gains, and $\alpha_{mn}$ is the loss of the higher-order mode.

Under thermo-optic coupling, the HOM may experience net amplification over extended portions of the length of the fiber. When this occurs, the coupled equations predict an exponential growth in HOM power, which characterizes the onset of TMI.

### 2.9. Microscopic STRS Threshold and Connection with TMI

STRS provides the microscopic mechanism that triggers the coherent transfer of power between ${\mathrm{LP}}_{01}$ and higher-order modes [7,13,33]. The threshold occurs when the effective gain of the ${\mathrm{LP}}_{mn}$ mode, driven by the real part of the coupling coefficient $\chi_{mn}$, becomes positive over a significant portion of the fiber [17].

In the high-gain regime, where the fundamental mode dominates the propagation and the initial HOM power is very small, integration of the coupled equations leads to a simple analytical expression for the threshold power [13]:

(30) $$P_{01}^{\mathrm{th}}=\frac{1}{\chi_{mn}^{\prime }}\ln\;\left(x\;\frac{P_{01}\left(0\right)}{P_{mn}\left(0\right)}\right),$$

where $x=P_{mn}\left(L\right)/P_{01}\left(L\right)$ represents the fraction of HOM power at the fiber output used to define the threshold (typically x∼1–5%). In this regime, Re{χmn} appears as the dominant parameter setting the threshold, while the ratio $P_{01}\left(0\right)/P_{mn}\left(0\right)$ controls the sensitivity to the initial HOM fraction.

This expression shows that, in large-mode-area fibers, the threshold depends primarily on the thermo-optic efficiency encapsulated in Re{χmn}, becoming weakly influenced by the total gain when the product $g_{01}L$ is sufficiently large [13,33]. Under moderate-gain conditions, HOM losses and gain nonuniformities increase the threshold; here, such effects are incorporated directly through numerical integration of the propagation equations.

The STRS threshold therefore defines the microscopic scale at which the higher-order mode begins to grow exponentially due to thermal coupling. TMI emerges when this growth manifests macroscopically at the amplifier output, altering the beam profile and degrading its quality [21,32].

### 2.10. Analytical Estimate of the TMI Threshold

The macroscopic TMI threshold is reached when small transverse perturbations begin to grow exponentially and transfer power from the fundamental mode to HOMs. Among the available analytical approaches, the formulation of Zervas [38,39] provides a compact expression that relates the thermal threshold to the optical, thermal, and geometrical parameters of the fiber.

In the regime typical of high-power amplifiers, the reduced threshold expression can be written as follows [38,39]:

(31) $$P_{\mathrm{TMI}}^{\mathrm{thr}}=\frac{\kappa_{0}\;U_{mn}^{2}\;\left(U_{mn}^{2}-U_{01}^{2}\right)}{4\pi \;n_{\mathrm{eff}}\left(\frac{\partial n}{\partial T}\right)\alpha_{s}^{\prime }}{\left(\frac{\lambda_{s}}{D_{0}}\right)}^{2},$$

where $\kappa_{0}$ is the thermal conductivity of silica; $U_{01}$ and $U_{mn}$ are the transverse wavenumbers of the fundamental and coupled modes, respectively; and $D_{0}$ is the diameter of the core.

The quantity $\alpha_{s}^{\prime }$ represents an effective loss of the fundamental mode, incorporating both intrinsic losses and the dissipative term associated with the quantum defect. According to the formulation developed by Zervas [38], this loss is written in terms of a saturated gain of the fundamental mode:

(32) $$\alpha_{s}^{\prime }\;≃\;\alpha_{s}+\frac{1}{2}\;q_{D}\;g_{s},$$

where $\alpha_{s}$ is the background loss (including photodarkening), and $q_{D}$ is the quantum defect. In the context of modern STRS modeling, $g_{s}$ must be associated with the effective STRS gain introduced by Dong rather than the small-signal gain $g_{0}$. Dong showed that the heating relevant to STRS can be written as follows [13,36]:

(33) $$\tilde{Q}\left(\vec{r},z\right)=\left(\frac{\lambda_{s}}{\lambda_{p}}-1\right)\tilde{g}\left(\vec{r},z\right)\;\tilde{I}\left(\vec{r},z\right),$$

where $\tilde{g}\left(\vec{r},z\right)$ is the *effective STRS gain*, which is given by

(34) $$\tilde{g}\left(\vec{r},z\right)=\left[\left(\sigma_{es}+\sigma_{as}\right)\;{\tilde{n}}_{2}\left(\vec{r},z\right)\;\left(1-\eta \left(\vec{r},z\right)\right)-\sigma_{as}\right]\;N_{0}.$$

${\tilde{n}}_{2}$ is the normalized inversion obtained from the rate equations, and η is the local conversion efficiency for the signal. To reconcile the expression developed by Zervas with this formalism, in this work, we set

(35) $$g_{s}\left(z\right)=\frac{\int_{0}^{a}\tilde{g}\left(r,z\right)\;f_{01}^{2}\left(r\right)\;r\;dr}{\int_{0}^{a}f_{01}^{2}\left(r\right)\;r\;dr},$$

That is, $g_{s}$ is the effective STRS gain weighted by the modal profile of the fundamental mode in the core. The additional term $\left(1/2\right)\;q_{D}\;g_{s}$ in Equation (32) therefore reflects the fraction of the amplified power that is converted into heat by the combination of the quantum defect and the saturated effective gain that governs STRS and the onset of instability.

Equation (31) correctly captures the dependence of the TMI threshold on mode size, the thermo-optic response of the glass, and the thermal efficiency of the material. Although it poses quantitative limitations for more complex fiber geometries, it remains a useful design reference, enabling direct interpretation of how thermal and optical parameters influence the emergence of instability.

## 3. Results and Discussion

Numerical simulations of a Yb-DCF amplifier were performed in MATLAB (R2016a) using the geometrical, optical, thermal, and active-medium parameters summarized in Table 1. For comparison purposes, an SCF configuration was also considered using the same geometrical dimensions (a=12.5μm/b=200μm).

The small-signal pump absorption coefficient at $\lambda_{p}=976\;\mathrm{nm}$ follows directly from the pump propagation equation in the limit $N_{2}\ll N$, $\alpha_{p,\mathrm{ss}}=\Gamma_{p}\sigma_{p}^{a}N_{\mathrm{Yb}}+\alpha_{p}$ [40,41], yielding $\alpha_{p,\mathrm{ss}}=0.36\;m^{-1}$ for the parameters listed in Table 1. The pump power $P_{p}^{+}\left(0\right)=2\;\mathrm{kW}$ represents the net optical power coupled with the inner cladding at the fiber input end; insertion losses of pump diodes and coupling optics are therefore not explicitly modeled. The system analyzed in this work corresponds to a forward-pumped fiber amplifier, and no optical resonant cavity is involved. In the present forward-pumped amplifier configuration, no counter-propagating pump component is considered. The active fiber length is L=12m, and the amplifier operates at fixed pump and signal wavelengths ($\lambda_{p}=976\;\mathrm{nm}$, and $\lambda_{s}=1064\;\mathrm{nm}$), so spectral scanning is outside the scope of the present continuous-wave (CW) model.

The model integrates modal propagation, quantum-defect heating, and the thermal–optic interaction described by STRS in a three-layer Yb-DCF under forward pumping.

This framework provides a self-consistent description of the axial evolution of modal power, the radial temperature distribution, and the nonlinear coupling between ${\mathrm{LP}}_{01}$ and higher-order modes. A central aspect of the analysis is the role of the inner cladding: its presence modifies the optical distribution of the modes and the thermal diffusion pathways, thereby altering the overlap integrals that govern STRS-induced modal coupling.

The following subsections examine how the multi-layer geometry affects the temperature field generated by the quantum defect, the axial dependence of the coupling coefficient $\chi_{mn}\left(z\right)$, and the resulting modal power transfer. For completeness, the results are also compared with a single-clad fiber with an identical core radius and numerical aperture. This contrast highlights how the transverse architecture—through its influence on modal eigenvalues, confinement, and thermal redistribution—determines the conditions for the onset and evolution of transverse mode instability.

### 3.1. Pump and Signal Power Evolution

**Figure 2:**
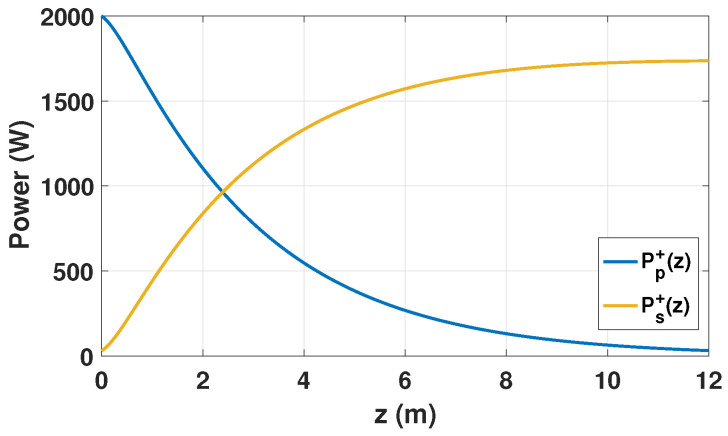
Simulated axial evolution of the forward pump power $P_{p}^{+}\left(z\right)$ and forward signal power $P_{s}^{+}\left(z\right)$ along the Yb-DCF in a forward-pumped fiber amplifier.

Figure 2 shows the axial evolution of the pump and signal power along the Yb-DCF that forms the active medium of the amplifier schematically illustrated in Figure 1. The pump is injected only at the fiber input end (z=0) with the launched power $P_{p}^{+}\left(0\right)$ defined in Table 1. The amplifier is seeded at z=0 with an input signal power $P_{s}^{+}\left(0\right)=30\;W$, and the output signal power is given by the forward signal at the fiber end, $P_{\mathrm{out}}=P_{s}^{+}\left(L\right)$.

**Figure 3:**
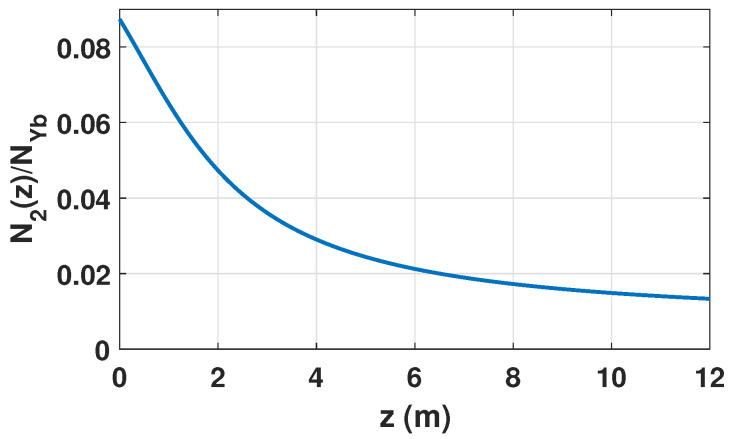
Simulated axial distribution of the normalized population inversion $N_{m}\left(z\right)=N_{2}\left(z\right)/N_{Yb}$ along the Yb-DCF under forward pumping.

As the pump is absorbed by the active medium, the forward pump component $P_{p}^{+}\left(z\right)$ decreases monotonically along the fiber, while the forward signal component $P_{s}^{+}\left(z\right)$ increases monotonically due to stimulated emission under gain-saturated conditions. In the absence of counter-propagating pump and signal waves, the resulting axial inversion profile is solely determined by the longitudinal evolution of the forward pump and signal power, as shown in Figure 3.

This axial inversion profile defines the spatial distribution of quantum-defect heating along the fiber, which constitutes the driving mechanism for STRS and the thermally induced modal coupling discussed in the following sections.

For the operating conditions considered here, the forward signal power increases from the injected seed level $P_{s}^{+}\left(0\right)=30\;W$ to an output value of $P_{\mathrm{out}}=P_{s}^{+}\left(L\right)=1.736\;\mathrm{kW}$ at the fiber end. The corresponding optical efficiency of the forward-pumped fiber amplifier is defined as follows:

(36) $$\eta_{\mathrm{opt}}=\frac{P_{\mathrm{out}}-P_{s}^{+}\left(0\right)}{P_{p}^{+}\left(0\right)}.$$

For a launched pump power of $P_{p}^{+}\left(0\right)=2.0\;\mathrm{kW}$, this yields $\eta_{\mathrm{opt}}=85\%$, which remains below the quantum-defect limit given by $\lambda_{p}/\lambda_{s}\approx 0.92$.

### 3.2. Modal Field Distribution in SCF and DCF

**Figure 4:**
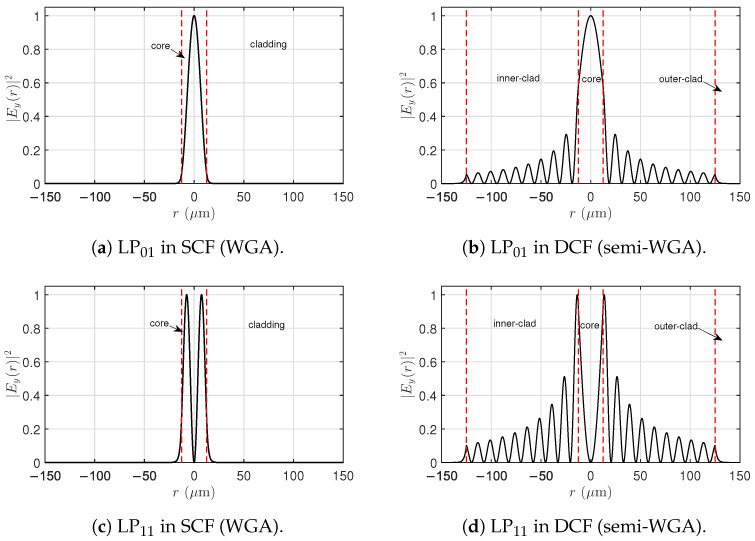
Normalized radial intensity distributions of the ${\mathrm{LP}}_{01}$ and ${\mathrm{LP}}_{11}$ modes in (**a**,**c**) single-clad and (**b**,**d**) double-clad fibers. The red dashed vertical lines mark the radial interfaces between the fiber layers, corresponding to the core and cladding in the SCF and to the core, inner cladding, and outer cladding in the DCF. In SCFs, both modes remain tightly confined to the core, whereas in DCFs, penetration into the inner cladding is observed due to the three-layer structure.

Figure 4 compares the radial distributions of the ${\mathrm{LP}}_{01}$ and ${\mathrm{LP}}_{11}$ modes in single-clad and double-clad fibers. In the SCF, both modes remain tightly confined to the core, as expected from a two-layer step-index structure in which no intermediate lower-index region is present. The absence of an inner cladding prevents significant field penetration beyond the core, resulting in higher modal concentration in the doped gain region.

In the DCF, the presence of the intermediate inner cladding modifies the modal structure, allowing penetration of the guided modes into the lower-index region. This redistribution spreads the optical power over a larger transverse area and reduces the fraction confined to the core. Such changes in modal localization are expected to influence both the effective refractive-index separations and the physical parameters that appear in threshold models for modal instability.

The quantitative impact of this modal restructuring on TMI is evaluated in the next subsection through the transverse eigenvalues and the corresponding instability thresholds.

### 3.3. Modal Eigenvalues and TMI Threshold

Identification of the dominant coupling channel is directly related to the transverse eigenvalues of the modes involved. For the double-clad fiber, the eigenvalues were computed using both the rigorous three-layer formulation developed by Tsao et al. [27] and the semi–weakly guiding model developed by Tan et al. [24]. These formalisms incorporate the influence of the inner cladding in different ways, resulting in distinct predictions for $U_{01}$ and $U_{mn}$. For the Tan semi–WGA formalism, the modal solutions were obtained under Condition 1 ($k_{0}n_{3}<\beta <k_{0}n_{2}$) [24]. As discussed in Ref. [24], because $n_{1}\approx n_{2}$ in typical DCF structures, the core-guided Condition 2 ($k_{0}n_{2}<\beta <k_{0}n_{1}$) can hardly be achieved, and the analytical development proceeds under Condition 1. In the same framework, WGA is applied only at the core–inner-cladding interface, whereas the inner–outer cladding interface retains the $n_{2}/n_{3}$ dependence (semi-WGA), since $n_{2}\approx n_{3}$ is not a valid assumption for most DCFs. For completeness, enforcing Condition 2 in our solver yields $U_{01}=1.9526$ and $U_{11}=3.0780$, illustrating the expected SCF-like limit when $n_{1}\approx n_{2}$ and confirming that the conventional core-guided regime is recovered when required. Since the asymptotic threshold expression Equation (31) depends explicitly on these eigenvalues, the choice of modal model directly affects the predicted instability behavior.

For comparison, a single-clad fiber with the same core radius and numerical aperture was analyzed using the classical scalar step-index dispersion relation developed by Gloge [18]. The resulting eigenvalues $U_{01}=1.9485$ and $U_{11}=3.0706$ reflect the stronger optical confinement characteristic of a two-layer structure.

Table 2 summarizes the modal eigenvalues and the corresponding threshold power obtained from Equation (31), assuming $\lambda_{s}=1064$ nm, $D_{0}=25\;\mu$m, and $q_{D}≃9\%$.

The results reveal a clear ordering of instability thresholds. When we use the rigorous eigenvalues developed by Tsao et al. [27], the ${\mathrm{LP}}_{01}$–${\mathrm{LP}}_{11}$ channel yields the lowest threshold (≈1.05 kW), while the ${\mathrm{LP}}_{01}$–${\mathrm{LP}}_{13}$ pair plays a secondary role with a significantly higher threshold. Tan’s semi-WGA model predicts that $U_{11}<U_{01}$ [24], which is not compatible with the ordering assumptions implicit in Equation (31). Therefore, within Equation (31), the ${\mathrm{LP}}_{01}$–${\mathrm{LP}}_{13}$ channel is the only applicable contribution in this case, yielding a threshold of ≈1.59 kW.

Although the SCF exhibits stronger optical confinement, its predicted threshold (≈2.18 kW) is higher than that of the DCF. In the asymptotic framework of Equation (31), this reflects the combined influence of (i) the transverse optical eigenvalues $U_{01}$ and $U_{mn}$, which differ substantially between two- and three-layer structures, and (ii) the thermal eigenvalues $\eta_{ml}$ associated with radial heat diffusion. For the present parameter regime, the contrast between SCF and DCF thresholds is dominated by the optical eigenvalues, while the thermal response provides a secondary yet consistent contribution to the overall STRS-driven coupling.

Overall, these comparisons demonstrate that the dominant TMI channel and the resulting threshold depend sensitively on both the modal formalism and the multi-layer waveguide geometry through their joint impact on the optical and thermal eigenvalue structure entered into Equation (31). Accurate prediction of TMI therefore requires internal consistency between the optical modal analysis, the thermal diffusion model, and the STRS-based asymptotic threshold.

### 3.4. Radial and Axial Temperature Profiles in the Yb-DCF

**Figure 5:**
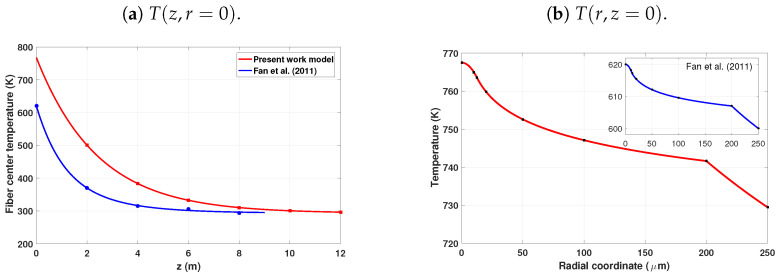
Temperature distributions in the active fiber: (**a**) axial evolution of the center temperature (r=0) and (**b**) radial profile at the amplifier input (z=0). The present 2kW forward-pumped Yb-DCF amplifier model (red) is compared with the results reported by Fan et al. [34], corresponding to Figure 3 and to the $h=100\;\;W\;m^{-2}\;K^{-1}$ curve in Figure 4 of Ref. [34], respectively.

Figure 5 shows the thermal behavior along the active fiber (Yb-DCF) in a forward-pumped Yb-DCF amplifier operating in the CW steady state, with $P_{p}\left(0\right)=2\;\;\mathrm{kW}$ and a seed power $P_{s}\left(0\right)=30\;\;W$. At the fiber input (z=0), the pump power is maximum, and heat deposition is strongest, resulting in a core/center temperature of T(z=0,r=0)=767.5K. As shown in Figure 5a, the temperature decreases monotonically with an increase in *z* due to pump absorption.

At the fiber output (z=L=12m), the remaining pump power is $P_{p}\left(12\;\;m\right)=30.92\;\;W$, while the forward signal reaches $P_{s}^{+}\left(12\;\;m\right)=1.74\;\;\mathrm{kW}$. The strong pump depletion and efficient optical conversion explain the progressive reduction in heat generation toward the fiber end, consistent with Figure 5a.

Table 3 lists representative axial core-center temperatures T(z,r=0) extracted from the curves shown in Figure 5a, allowing a direct quantitative comparison between the formulation in question and the results reported by Fan et al. [34]. The quantitative agreement along the fiber axis confirms the consistency of the thermal implementation, while the higher peak temperature in the present case reflects the higher pump power.

Beyond the quantitative agreement along the fiber axis, the variations observed in the radial and axial profiles further characterize the thermal regime associated with the higher pump power. As observed in Figure 5a, the axial temperature variation at r=0 increases from approximately 324 K in the 1.22 kW case (Fan et al.) to about 457 K in the present 2 kW regime. Similarly, from Figure 5b, the radial temperature difference between the core center and the cladding boundary at z=0 increases from approximately 20 K to about 38 K. These results indicate a rise in the thermal load in both the transverse and longitudinal directions, while the monotonic radial and axial behavior of the temperature distribution is maintained.

In silica-based double-clad fibers, the core and inner cladding exhibit similar thermal conductivities, whereas the outer polymer jacket has a significantly lower value [30,35]. Thus, within the glass region, the temperature profile is primarily determined by heat deposition in the doped core and by the thermal boundary condition at r=c. The behavior observed in Figure 5 therefore follows the axial pump-absorption pattern rather than strong thermal contrasts between the silica layers. The predicted temperature levels and thermal gradients are consistent with experimentally reported values for high-power Yb-doped fiber amplifiers operating under comparable cooling conditions [34], supporting the physical consistency of the thermal model adopted in this work. With respect to TMI, the inner cladding affects the coupling mechanism through the thermal eigenvalues and modes associated with the three-layer geometry, a finding consistent with the formulation in the previous section. For the parameter regime considered here, however, the dominant contrast between SCF and DCF thresholds originates from the transverse optical eigenvalues $U_{01}$ and $U_{mn}$ entered into Equation (31), while the thermal response provides the thermo-optic background that contributes to the STRS-driven coupling coefficient and the corresponding instability threshold.

### 3.5. STRS-Induced Modal Coupling

**Figure 6:**
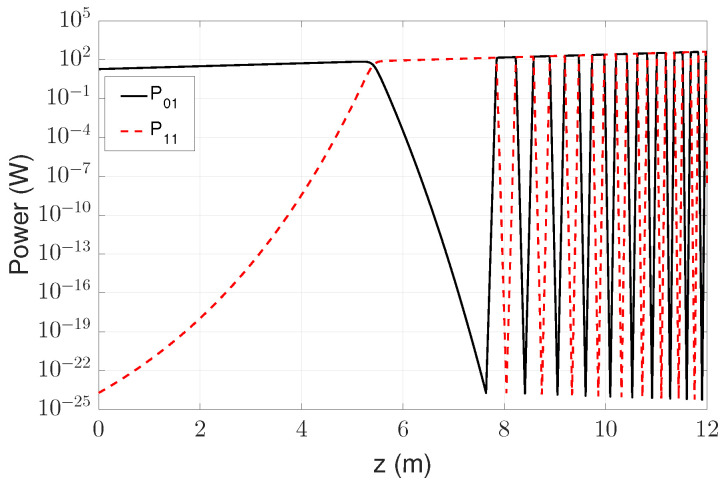
Simulated axial evolution of the modal power $P_{01}\left(z\right)$ and $P_{11}\left(z\right)$ in a Yb-DCF, showing the dominance of the fundamental mode in the first half of the fiber, the abrupt power transfer associated with the TMI threshold, and the subsequent oscillations resulting from STRS re-seeding.

Figure 6 shows the axial evolution of the modal power $P_{01}\left(z\right)$ and $P_{11}\left(z\right)$ along the fiber length (0≤z≤L). The curves were obtained by integrating the coupled differential equations, Equations (28) and (29), using the nonlinear thermo-optic coupling coefficient $\chi_{mn}\left(z,\Omega \right)$, which incorporates the radial thermal modes and eigenvalues.

From the input end up to approximately z≈L/2, the ${\mathrm{LP}}_{01}$ mode remains dominant and varies smoothly, whereas the ${\mathrm{LP}}_{11}$ mode, initially seeded at an extremely low noise floor, grows rapidly. In the simulations, a seed ratio of $r=P_{11}\left(0\right)/P_{01}\left(0\right)=10^{-25}$ was adopted, consistent with the deterministic STRS simulations reported by Dong [13]. Smith and Smith [42] showed that higher-order modes are inevitably seeded by quantum noise (with approximately one photon per beat cycle) as well as by scattering at fiber imperfections and the pump or signal. More recent modeling studies have further indicated that amplitude noise of quantum or relative-intensity-noise origin provides a realistic physical source for the ${\mathrm{LP}}_{11}$ seed participating in STRS [43]. The adopted seed level therefore represents a physically realistic background perturbation that initiates the STRS process. Additionally, because the instability threshold depends only logarithmically on the seed ratio, variations of over several orders of magnitude produce only weak changes in the predicted threshold. The modal fraction,

(37) $$\eta \left(z\right)=\frac{P_{11}\left(z\right)}{P_{01}\left(z\right)+P_{11}\left(z\right)}$$

therefore remains close to zero near the fiber input and increases significantly as *z* approaches L/2.

As the total power increases, the thermo-optic modulation of the refractive index forms a traveling thermal grating that enhances modal coupling. The instability threshold may be estimated directly from Equation (30). Introducing the seed ratio $r=P_{mn}\left(0\right)/P_{01}\left(0\right)$ transforms the expression into

(38) $$P_{01}^{\mathrm{th}}=\frac{\ln(1/x)-\ln r}{ℜ\{\chi_{mn}\}},$$

where x≃5% defines the fractional power criterion used to identify the onset of thermal instability.

For representative values $ℜ\left\{\chi_{11}\right\}\approx 1.3\times 10^{-1}\;\;W^{-1}$ and $r≃10^{-25}$, consistent with the parameters of the case shown in Figure 6, Equation (38) yields $P_{01}^{\mathrm{th}}=430\;\;W$. This value is in good agreement with the numerical threshold $P_{\mathrm{th}}=428.3\;\;W$ reported by Dong for the same amplifier configuration [13].

It is important to note that the threshold defined by Equation (30) does not correspond to the condition $P_{01}=P_{11}$ but rather to the point at which the modal fraction reaches η=x≃0.05, following Dong’s convention [13]. Accordingly, TMI is identified by the rapid rise in $P_{11}\left(z\right)$ and the associated decrease in $P_{01}\left(z\right)$, even when ${\mathrm{LP}}_{11}$ still carries only a small fraction of the total power.

**Figure 7:**
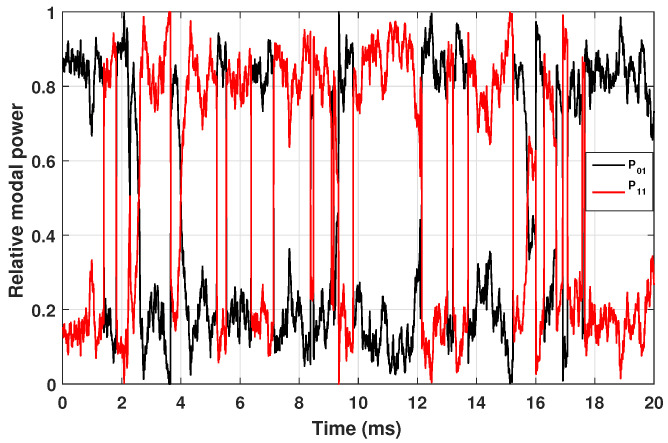
Numerically simulated temporal evolution of the relative modal power of the fundamental mode ${\mathrm{LP}}_{01}$ (black curve) and the higher-order mode ${\mathrm{LP}}_{11}$ (red curve) at the amplifier output after the onset of TMI. The intermittent energy exchange between the two transverse modes reflects the time-domain manifestation of STRS-induced modal coupling, whose axial build-up between ${\mathrm{LP}}_{01}$ and ${\mathrm{LP}}_{11}$ is shown in Figure 6.

While Figure 6 illustrates the spatial build-up of the STRS-driven modal coupling along the fiber length between the ${\mathrm{LP}}_{01}$ and ${\mathrm{LP}}_{11}$ modes, Figure 7 represents its corresponding time-domain signature at the output of the amplifier. The apparent irregular power exchange between the fundamental mode ${\mathrm{LP}}_{01}$ (FM) and the higher-order mode ${\mathrm{LP}}_{11}$ (HOM) is therefore a direct consequence of the axial thermo-optic coupling mediated by the traveling thermal grating. Such temporal modal power fluctuations directly translate into output-beam instability over time, which constitutes a hallmark of TMI in high-power fiber amplifiers, in agreement with the experimental observations reported by Jauregui et al. [10].

Above the threshold, the STRS dynamics become nonlinear. Energy may flow intermittently back into the fundamental mode, giving rise to an oscillatory behavior along the fiber. This effect is consistent with the “re-seeding” mechanism described by Dong [13], in which the traveling thermal grating continuously modulates the coupling direction according to the evolving modal power ratio. The resulting sequence of oscillations observed in the simulation reflects this dynamic feedback process.

### 3.6. Modal Coupling and TMI Evolution

**Figure 8:**
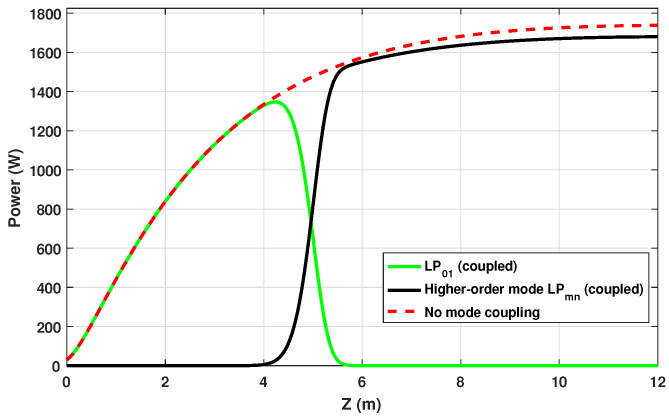
Axial evolution of the modal power $LP_{01}$ and $LP_{11}$ along the Yb-DCF. The dashed curve represents $LP_{01}$ in the absence of modal coupling. A pronounced STRS-induced power transfer can be observed around z=5m, when $P_{01}=730\;W$, as a result of the STRS mechanism, leading to the onset of TMI.

Figure 8 shows the axial evolution of modal power along the Yb-DCF, obtained from the complete amplifier model, assuming a seed power of $P_{01}\left(0\right)=30\;W$ at $\lambda_{s}=1064\;\mathrm{nm}$. The dashed curve corresponds to the reference case without modal coupling: the fundamental mode $LP_{01}$ grows monotonically over the 12 m solely under optical gain, indicating purely single-mode operation.

When thermo-optic coupling induced by STRS is included, the dynamics change qualitatively. Part of the power is transferred from $LP_{01}$ to a higher-order mode $LP_{11}$, as indicated by the coupled curves in Figure 8. This process is evidenced by the sharp drop in $P_{01}\left(z\right)$ at z∼5m, where the fundamental mode reaches $P_{01}\left(z\right)=730\;W$. From this point on, $LP_{11}$ grows rapidly and becomes the dominant mode.

Physically, this behavior corresponds to the onset of TMI: the thermal grating generated by quantum-defect heating produces a nonlinear coupling coefficient with sufficient magnitude and phase delay to invert the local gain balance between the modes. The initial noise component of $LP_{11}$ is coherently amplified, and power is progressively transferred from $LP_{01}$ to $LP_{11}$. The axial location of the threshold and the associated power level agree with the analytical prediction of Equation (30), reinforcing the interpretation of TMI as a thermo-optic instability driven by STRS.

The threshold behavior is qualitatively consistent with that reported for fiber amplifiers in [7]. In their model, the frequency-shifted interference between modes creates a moving irradiance pattern whose phase-lagged thermal response produces coherent modal coupling. The local small-signal gain g(z,Δν) of the higher-order mode is integrated to give $G\left(z\right)=\int_{0}^{z}g\left(z^{\prime }\right)\;dz^{\prime }$, resulting in $P_{2}\left(z\right)=P_{2}\left(0\right)e^{G(z)}$, with maximum gain occurring in the first half of the fiber (see Figures 7–11 of [7]).

In both models, the instability threshold appears as an abrupt modal transition triggered by thermally driven refractive-index modulation. In the present amplifier, this occurs at the point where the uncoupled $LP_{01}$ solution would otherwise continue to grow toward its nominal output value of $P_{01}\left(L\right)=1.74\;\mathrm{kW}$. Beyond the local threshold at z=5m, the higher-order mode becomes dominant, degrading beam quality and representing the macroscopic manifestation of TMI in continuous-wave high-power operation.

### 3.7. Modal Profiles, STRS-Driven Superposition, and Transverse Instability

**Figure 9:**
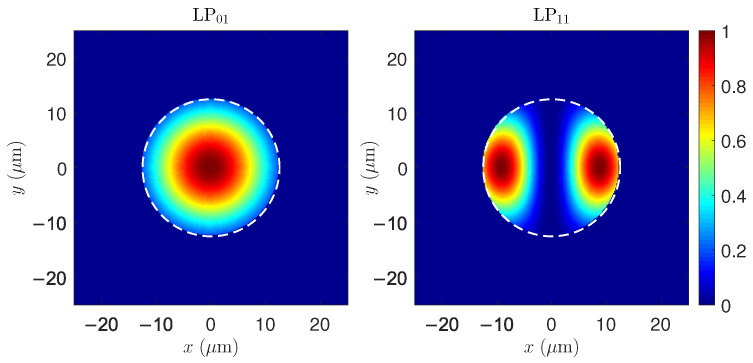
Two-dimensional maps of the normalized intensity of the ${\mathrm{LP}}_{01}$ and ${\mathrm{LP}}_{11}$ modes in the cross-section of the SCF. The central maximum of ${\mathrm{LP}}_{01}$ and the nodal pattern of ${\mathrm{LP}}_{11}$ are clearly visible.

Figure 9 shows the normalized transverse profiles of the ${\mathrm{LP}}_{01}$ and ${\mathrm{LP}}_{11}$ modes in the SCF. While ${\mathrm{LP}}_{01}$ exhibits a circularly symmetric central maximum, ${\mathrm{LP}}_{11}$ presents two characteristic lateral lobes separated by a nodal line. These transverse structures form the basis for the interference patterns that later drive thermal gratings through STRS.

These two-dimensional maps complement the radial profiles previously shown in Figure 4, providing the full transverse structure of the guided modes that participate in STRS and, ultimately, TMI.

**Figure 10:**
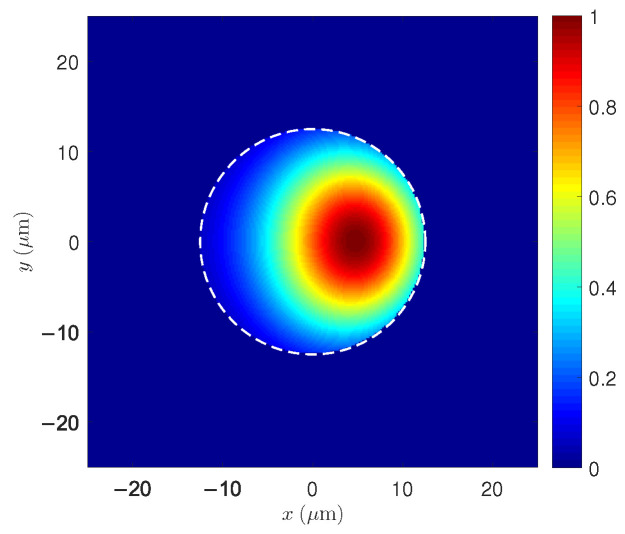
Transverse intensity pattern resulting from the normalized superposition of ${\mathrm{LP}}_{01}$ and ${\mathrm{LP}}_{11}$ during TMI in the SCF. The high spatial contrast in the core arises from thermal coupling induced by STRS, favoring power transfer into ${\mathrm{LP}}_{11}$.

Figure 10 shows the transverse normalized intensity pattern resulting from the superposition of ${\mathrm{LP}}_{01}$ and ${\mathrm{LP}}_{11}$ during TMI in the SCF. Unlike the isolated modes in Figure 9, the pattern here arises from a small ${\mathrm{LP}}_{11}$ component generated through the thermal–optic coupling induced by STRS. The strong optical confinement of both modes within the core produces high-contrast interference fringes, which modulate the heat generation associated with the quantum defect. This modulation reinforces the thermo-optic grating responsible for driving power transfer from ${\mathrm{LP}}_{01}$ to ${\mathrm{LP}}_{11}$.

**Figure 11:**
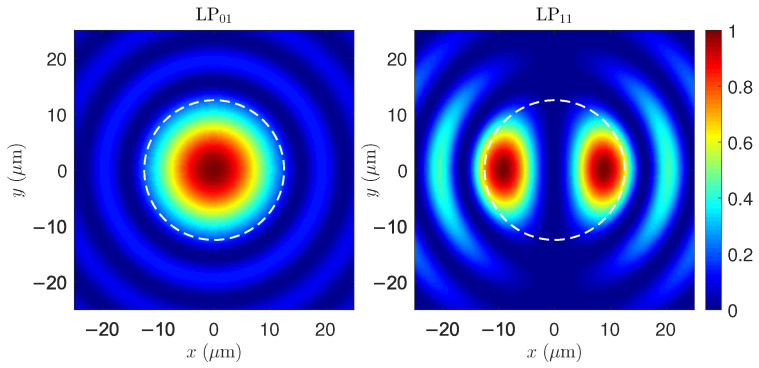
Normalized transverse modes of ${\mathrm{LP}}_{01}$ and ${\mathrm{LP}}_{11}$ in the DCF. Despite the radial extension of the fields into the inner cladding, the stronger thermal confinement enhances the thermo-optic grating, reducing the TMI threshold relative to the SCF.

Figure 11 shows the modes $LP_{01}$ and $LP_{11}$, again corresponding to the modes shown in Figure 4 in the DCF. Because the optical modes extend radially into the inner cladding, the interference pattern becomes broader and less concentrated near the fiber axis compared with the SCF. This spatial restructuring modifies the distribution of the thermo-optic source term associated with quantum-defect heating, altering the strength and shape of the resulting thermal grating. Although the thermal conductivities of the silica core and inner cladding are similar, the three-layer geometry changes the effective overlap between the optical interference pattern and the radial thermal modes. As a consequence, the STRS-induced coupling becomes more efficient in the DCF than in the SCF, consistent with the lower thresholds predicted from the transverse eigenvalues in the preceding sections.

**Figure 12:**
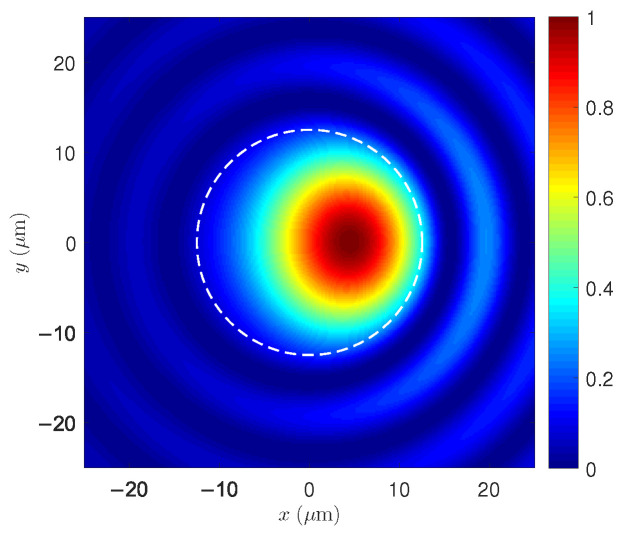
Axial evolution of the transverse patterns resulting from the STRS-driven coherent superposition of the ${\mathrm{LP}}_{01}$ and ${\mathrm{LP}}_{11}$ electric fields in the DCF. The periodic distortion synchronized with the growth of $P_{11}\left(z\right)$ reveals the modal transfer process characteristic of TMI.

Finally, Figure 12 shows the axial evolution of the transverse intensity patterns in the DCF during modal instability. These patterns result from the STRS-driven coherent superposition of the ${\mathrm{LP}}_{01}$ and ${\mathrm{LP}}_{11}$ electric fields. The periodic distortion of the intensity distribution, synchronized with the increase in $P_{11}\left(z\right)$, is a hallmark of STRS-driven modal coupling. The migration of optical power within the core region reflects the onset of TMI, providing a direct visualization of the underlying instability mechanism.

Taken together, these visualizations demonstrate that the onset and strength of STRS—and therefore robustness against TMI—are not governed solely by optical confinement. In the SCF, strong confinement produces high-contrast interference fringes, but the corresponding modal eigenvalues lead to a higher instability threshold. In the DCF, the three-layer geometry redistributes the modal fields and modifies their overlap with the thermal modes, resulting in a more effective STRS coupling mechanism and a lower TMI threshold. These observations are in agreement with the eigenvalue analysis and threshold predictions presented in the preceding sections.

### 3.8. Consistent Optical–Thermal Predictions of the TMI Threshold in SCF and DCF

The instability threshold was computed based on Equation (31) using the transverse eigenvalues for both the SCF and the DCF within the asymptotic STRS-based framework originally developed for high-power fiber amplifiers [38]. For the SCF, the eigenvalues obtained from the scalar step-index dispersion relation yield $U_{01}=1.9485$ and $U_{11}=3.0706$, corresponding to strong modal confinement in the core. For the DCF, the dispersion relations developed by Tsao et al. [27] predict the values listed in Table 1, reflecting the modified modal structure resulting from the three-layer geometry.

Assuming $D_{0}=25\;\mu$m and $\lambda_{s}=1064$ nm, the resulting quantum defect is $q_{D}\approx 9\%$ for a pump wavelength of $\lambda_{p}=976$ nm. Under these operating conditions, the predicted instability thresholds are $P_{\mathrm{TMI}}^{\mathrm{thr}}=2.18$ kW for the SCF and $P_{\mathrm{TMI}}^{\mathrm{thr}}=1.05$ kW for the DCF. Although the SCF supports a more tightly confined optical mode, the DCF exhibits a lower threshold. This apparent contradiction highlights that, within the asymptotic STRS model for amplifiers, the TMI threshold is not only governed by modal confinement but also by the combined effect of quantum-defect heating and the transverse eigenvalue structure. In the present model, this behavior is explained by the different transverse eigenvalues of the two waveguides: the three-layer structure of the DCF yields a smaller modal factor $U_{mn}^{2}\left(U_{mn}^{2}-U_{01}^{2}\right)$ in Equation (31), leading to a reduced asymptotic threshold by lowering the critical signal power required for STRS-driven mode coupling in the amplifier regime, despite its weaker optical confinement.

**Figure 13:**
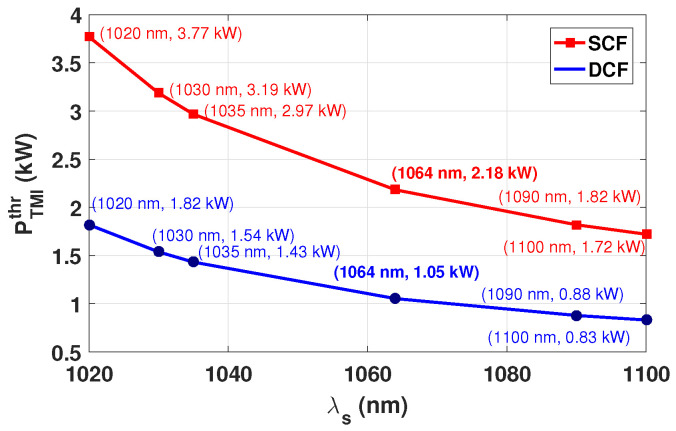
TMI threshold power $P_{\mathrm{TMI}}^{\mathrm{thr}}$ as a function of the quantum defect for SCF and DCF fibers. The annotated values along the SCF and DCF curves correspond to $P_{\mathrm{TMI}}^{\mathrm{thr}}$ evaluated at the indicated $\lambda_{s}$. For $\lambda_{s}=1064$ nm and $\lambda_{p}=976$ nm, the calculated thresholds at $D_{0}=25\;\mu$m are $P_{\mathrm{TMI}}^{\mathrm{thr}}=2.18$ kW (SCF) and $P_{\mathrm{TMI}}^{\mathrm{thr}}=1.05$ kW (DCF).

Figure 13 shows the dependence of the threshold on the quantum defect.

In connection with the spectral behavior shown in Figure 13, it should be noted that the behavior of $P_{\mathrm{TMI}}^{\mathrm{thr}}$ as a function of $\lambda_{s}$ is derived from the asymptotic TMI threshold formalism [38], in which the wavelength dependence enters primarily through the $q_{D}$ parameter and the transverse optical eigenvalue structure governing modal coupling. In contrast, the semi-analytical model reported by Tao et al. [36] includes wavelength-dependent gain saturation and spatial hole burning, which can produce non-monotonic spectral features with a maximum near 1030 nm. These effects were intentionally excluded in the present formulation. Despite the different modeling assumptions, the overall decrease in the TMI threshold toward longer signal wavelengths shown in Figure 13 remains qualitatively consistent with the trend reported in [36].

**Figure 14:**
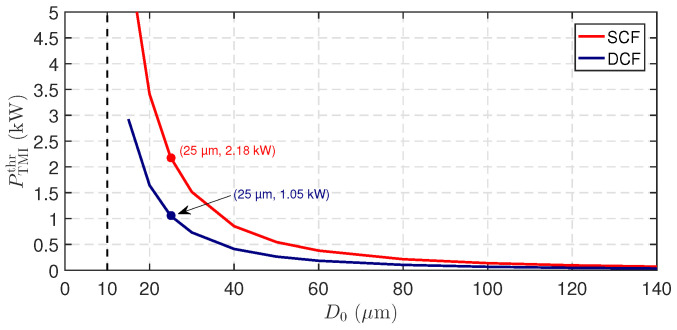
TMI threshold power $P_{\mathrm{TMI}}^{\mathrm{thr}}$ as a function of the core diameter $D_{0}$ for DCF and SCF fibers, computed using the Zervas model [21,38], with the same fiber and wavelength parameters used in Figure 13.

To confirm the consistency of these results, the threshold was also evaluated as a function of core diameter $D_{0}$ while maintaining the same operating wavelengths $\lambda_{s}=1064$ nm and $\lambda_{p}=976$ nm. As shown in Figure 14, the thresholds obtained at $D_{0}=25\;\mu$m match those derived from the quantum-defect parameterization. This agreement is expected since both formulations originate from the same asymptotic expression (31) and depend on the same transverse eigenvalue structure. In addition, to benchmark the present SCF predictions against published amplifier data, discrete threshold values extracted from the Zervas model are summarized in Table 4.

The quantum defect governs the longitudinal heat load, while the transverse eigenvalues determine how this heat distribution couples the ${\mathrm{LP}}_{01}$ mode with higher-order modes through STRS. Because the DCF exhibits a modified thermal diffusion profile associated with its three-layer geometry, the resulting temperature distribution interacts differently with the modal intensity patterns. Within the asymptotic framework proposed by Zervas for fiber amplifiers [38], this difference manifests primarily through the dependence of the threshold on the transverse eigenvalue structure appearing in Equation (31), while the local thermo-optic term remains governed by the thermal and saturation parameters of the model, such as ∂n/∂T and $\alpha_{s}^{\prime }$. Consequently, the lower threshold obtained for the DCF in both parameterizations is not incidental: it emerges from a unified optical–thermal mechanism linking quantum-defect heating, transverse modal structure, thermal diffusion, and the nonlinear coupling coefficient $\chi_{mn}$.

Table 4 compares the SCF TMI threshold values predicted by the amplifier-based model employed in the present work with those reported by Zervas [38], considering V=3 and a saturated gain coefficient $g_{s}=4.6\;\;m^{-1}$. The remaining fiber and amplifier parameters not explicitly specified in [38] were adopted from the simulation parameter set listed in Table 1. Small discrepancies are therefore expected due to differences in thermal boundary conditions and material parameters.

**Figure 15:**
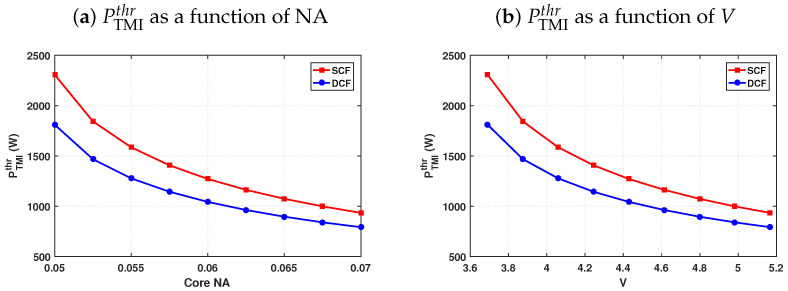
Dependence of $P_{\mathrm{TMI}}^{thr}$ on (**a**) NA and (**b**) *V* for SCF and DCF, calculated using the Zervas model. The observed trends reflect the influence of NA and *V* on the modal eigenvalue structure governing ${\mathrm{LP}}_{01}$–${\mathrm{LP}}_{11}$ coupling.

Figure 15 shows that the instability threshold’s dependence on the core numerical aperture (NA) and the normalized frequency parameter *V* is directly related to the modal structure supported by the fiber. Since the asymptotic threshold expression derived from the Zervas model [38] depends explicitly on the transverse eigenvalues $U_{01}$ and $U_{mn}$, variations in NA (and therefore *V*) modify the modal confinement and the eigenvalue separation governing the ${\mathrm{LP}}_{01}$–${\mathrm{LP}}_{mn}$ coupling responsible for STRS-driven TMI. In the present case, the dominant channel corresponds to the ${\mathrm{LP}}_{01}$–${\mathrm{LP}}_{11}$ pair, and the resulting NA- and *V*-dependent trends are qualitatively consistent with the numerical and experimental observations reported by Tao et al. [44], although the specific fiber and amplifier parameters considered here are not identical.

## 4. Conclusions

In this work, we developed and applied a coupled optical–thermal formulation for the analysis of TMI in high-power continuous-wave double-clad ytterbium-doped fiber amplifiers, explicitly accounting for the multi-layer radial geometry. The model incorporated modal profiles obtained from dispersion relations, radial thermal modes, gain saturation, and the nonlinear coupling coefficient $\chi_{mn}$ associated with stimulated thermal Rayleigh scattering (STRS). The interaction between the fundamental ${\mathrm{LP}}_{01}$ mode and higher-order modes was described through coupled power equations, reproducing both modal transfer and the onset of the thermal instability threshold.

The results show that replacing a two-layer SCF with a three-layer DCF alters the transverse modal eigenvalues and the associated optical–thermal overlap integrals. For the geometry investigated, the eigenvalue structure imposed by the inner cladding leads to stronger STRS-induced coupling and, consequently, a lower TMI threshold for the DCF, even though the silica-related thermal parameters are essentially the same in both fibers. A quantitative comparison with previously reported SCF results demonstrated good agreement with the literature over a wide range of core diameters, validating the present model and confirming the systematic reduction in the TMI threshold in DCFs. Eigenvalue analysis further confirmed that the dominant instability channel depends sensitively on the modal structure: the rigorous Tsao formalism predicted preferential coupling between ${\mathrm{LP}}_{01}$ and ${\mathrm{LP}}_{11}$, whereas the formulation of Tan yielded a physically meaningful threshold only for the ${\mathrm{LP}}_{01}$–${\mathrm{LP}}_{13}$ pair because of the relative ordering of the transverse eigenvalues. These differences highlight the importance of maintaining internal consistency between optical modal analysis and the thermal diffusion model when evaluating thermally induced modal instabilities.

In summary, the observed instability is a manifestation of thermally driven modal coupling in high-power fiber amplifiers: quantum-defect heating generates a delayed refractive-index modulation that forms a traveling thermal grating responsible for coherent intermodal power transfer. This mechanism provides a consistent physical interpretation of the STRS-based modal coupling described by Dong and by Smith & Smith while remaining compatible with the asymptotic thermal-instability threshold scaling predicted by the analytical model developed by Zervas. The threshold analysis for variations in the core NA showed that $P_{\mathrm{TMI}}^{\mathrm{thr}}$ adheres to an NA- and *V*-dependent behavior determined by changes in the transverse eigenvalues governing ${\mathrm{LP}}_{01}$–${\mathrm{LP}}_{11}$ coupling. As a direct implication of this modal requirement, in the single-mode regime (e.g., $D_{0}=10\;\mu$m), where no guided HOM exists, an FM–HOM TMI threshold associated with ${\mathrm{LP}}_{01}$–${\mathrm{LP}}_{11}$ power transfer is not defined within the adopted formulation. The numerical results further show that the axial build-up of STRS-induced coupling naturally gives rise to intermittent temporal energy exchange between transverse modes at the amplifier output, a finding that is in agreement with experimental observations of beam fluctuations reported in the literature. The formulation presented here quantifies the impact of multi-layer fiber geometry on thermo–optic coupling and provides a foundation for designing high-power fiber lasers with improved modal stability.

## Figures and Tables

**Table 1:** Physical, geometrical and laser parameters used in the numerical simulations.

Symbol	Parameter	Value
Fiber geometry and refractive indices		
*a*	Core radius	12.5 μm
*b*	Inner-cladding radius	125 μm
*c*	Outer-cladding radius	200 μm
$n_{1}$	Core refractive index	1.446612
$n_{2}$	Inner-cladding refractive index	1.445367
$n_{3}$	Outer-cladding refractive index	1.370214
NA	Numerical aperture	0.06
*L*	Active fiber length	12 m
Optical parameters		
$\lambda_{p}$	Pump wavelength	976 nm
$\lambda_{s}$	Signal wavelength	1064 nm
*V*	Normalized frequency	4.43
$n_{\mathrm{eff}}$	Effective index (${\mathrm{LP}}_{01}$)	1.446548
Yb^3+^ active medium		
$N_{\mathrm{Yb}}$	Yb^3+^ concentration	$6\times 10^{25}\;m^{-3}$
$\tau_{2}$	Upper-state lifetime	1 ms
$\sigma_{p}^{a}$	Pump absorption cross section	$6\times 10^{-25}\;m^{2}$
$\sigma_{p}^{e}$	Pump emission cross section	$2.5\times 10^{-27}\;m^{2}$
$\sigma_{s}^{a}$	Signal absorption cross section	$1.4\times 10^{-27}\;m^{2}$
$\sigma_{s}^{e}$	Signal emission cross section	$2.0\times 10^{-25}\;m^{2}$
$\Gamma_{s}$	Signal filling factor	0.82
$\Gamma_{p}$	Pump filling factor	0.01
Thermal parameters		
$\kappa_{0}$	Thermal conductivity	1.38W/(mK)
ρ	Material density	$2200\;\mathrm{kg}/m^{3}$
*C*	Specific heat capacity	703J/(kgK)
*D*	Thermal diffusivity	$8.9\times 10^{-7}\;m^{2}/s$
dn/dT	Thermo-optic coefficient	$1.2\times 10^{-5}\;K^{-1}$
$T_{c}$	Ambient temperature	298 K
Pump and signal		
$P_{p}^{+}\left(0\right)$	Forward pump power	2 kW
$P_{s}\left(0\right)$	Seed signal power	30 W
$\alpha_{p}$	Pump loss	$3.0\times 10^{-3}\;m^{-1}$
$\alpha_{s}$	Signal loss	$5.0\times 10^{-3}\;m^{-1}$

**Table 2:** Transverse eigenvalues and TMI threshold power $P_{\mathrm{TMI}}^{\mathrm{thr}}$ at $\lambda_{s}=1064$ nm, as obtained from Equation (31).

Mode Pair	$U_{01}$	$U_{\mathrm{mn}}$	$P_{\mathrm{TMI}}^{\mathrm{thr}}$ (kW)
DCF–Tsao et al.			
${\mathrm{LP}}_{01}$–${\mathrm{LP}}_{11}$	1.616	2.561	1.05
${\mathrm{LP}}_{01}$–${\mathrm{LP}}_{13}$	1.616	4.358	8.63
DCF–Tan et al. (Condition 1)			
${\mathrm{LP}}_{01}$–${\mathrm{LP}}_{11}$	1.0159	0.7824	not applicable ($U_{11}<U_{01}$)
${\mathrm{LP}}_{01}$–${\mathrm{LP}}_{13}$	1.0159	2.5992	1.59
SCF–Gloge			
${\mathrm{LP}}_{01}$–${\mathrm{LP}}_{11}$	1.9485	3.0706	2.18

**Table 3:** Core-center temperature T(z,r=0) at selected axial positions. Fan et al.’s data were reproduced from Ref. [34].

*z* (m)	T(z,r=0) (K)—Fan et al. [34]	T(z,r=0) (K)—Present Work
0	620.01	767.50
2	369.79	500.13
4	316.36	383.42
6	301.41	332.47
8	296.27	310.23

**Table 4:** Comparison of TMI threshold power for SCF as a function of core diameter $D_{0}$.

$D_{0}$ (μm)	$P_{\mathrm{TMI}}^{\mathrm{thr}}$ (kW)—This Work	$P_{\mathrm{TMI}}^{\mathrm{thr}}$ (kW)—[38]
20	1.97	1.74
25	1.26	1.08
30	0.88	0.79
35	0.65	0.60
40	0.49	0.49

## Data Availability

The original contributions presented in this study are included in the article. Further inquiries can be directed to the corresponding author.

## References

[1] Yan P., Wang X., Li D., Huang Y., Sun J., Xiao Q., Gong M. (2017). High-power 1018 nm ytterbium-doped fiber laser with output of 805 W. Opt. Lett..

[2] Xiao Y., Brunet F., Kanskar M., Faucher M., Wetter A., Holehouse N. (2012). 1-kilowatt CW all-fiber laser oscillator pumped with wavelength-beam-combined diode stacks. Opt. Express.

[3] Jauregui C., Limpert J., Tünnermann A. (2013). High-power fibre lasers. Nat. Photonics.

[4] Zhou P., Xiao H., Leng J., Xu J., Chen Z., Zhang H., Liu Z. (2017). High-power fiber lasers based on tandem pumping. J. Opt. Soc. Am. B.

[5] Yan P., Wang X., Wang Z., Huang Y., Li D., Xiao Q., Gong M. (2018). A 1150-W 1018-nm fiber laser bidirectional pumped by wavelength-stabilized laser diodes. IEEE J. Sel. Top. Quantum Electron..

[6] Cardoso E.S., Samad R.E., Motta C.C. Refractive index change analysis in a high-power Yb-doped double-clad fiber laser. Proceedings of the 2021 SBFoton International Optics and Photonics Conference (SBFoton IOPC).

[7] Smith A.V., Smith J.J. (2011). Mode instability in high power fiber amplifiers. Opt. Express.

[8] Jauregui C., Eidam T., Otto H.J., Stutzki F., Jansen F., Limpert J., Tünnermann A. (2012). Physical origin of mode instabilities in high-power fiber laser systems. Opt. Express.

[9] Zervas M.N., Codemard C.A. (2014). High power fiber lasers: A review. IEEE J. Sel. Top. Quantum Electron..

[10] Jauregui C., Stihler C., Limpert J. (2020). Transverse mode instability. Adv. Opt. Photonics.

[11] Cardoso E.S., Samad R.E., Motta C.C. Comparative Analysis of Transverse Mode Instability in Single-Clad and Double-Clad Fibers. Proceedings of the 2025 SBFoton International Optics and Photonics Conference (SBFoton IOPC).

[12] Smith A.V., Smith J.J. (2013). Spontaneous Rayleigh seed for stimulated Rayleigh scattering in high power fiber amplifiers. IEEE Photonics J..

[13] Dong L. (2013). Stimulated thermal Rayleigh scattering in optical fibers. Opt. Express.

[14] Kong F., Xue J., Stolen R.H., Dong L. (2016). Direct experimental observation of stimulated thermal Rayleigh scattering with polarization modes in a fiber amplifier. Optica.

[15] Tao R., Wang X., Zhou P. (2018). Comprehensive theoretical study of mode instability in high-power fiber lasers by employing a universal model and its implications. IEEE J. Sel. Top. Quantum Electron..

[16] Dong L., Ballato J., Kolis J. (2023). Power scaling limits of diffraction-limited fiber amplifiers considering transverse mode instability. Opt. Express.

[17] Dong L., Zervas M. (2023). Transverse mode instability in fiber laser oscillators. Opt. Express.

[18] Gloge D. (1971). Weakly guiding fibers. Appl. Opt..

[19] Li J., Duan K., Wang Y., Cao X., Zhao W., Guo Y., Lin X. (2008). Theoretical analysis of the heat dissipation mechanism in Yb^3+^-doped double-clad fiber lasers. J. Mod. Opt..

[20] Naderi S., Dajani I., Madden T., Robin C. (2013). Investigations of modal instabilities in fiber amplifiers through detailed numerical simulations. Opt. Express.

[21] Zervas M.N. (2019). Transverse mode instability, thermal lensing and power scaling in Yb^3+^-doped high-power fiber amplifiers. Opt. Express.

[22] Dong L. (2023). Transverse mode instability considering bend loss and heat load. Opt. Express.

[23] Li H., Huang L., Wu H., Wang X., Zhou P. (2024). Simplified expression for transverse mode instability threshold in high-power fiber lasers. Opt. Express.

[24] Tan X., Liu X., Zhao W., Li C., Wang Y., Li J. (2013). Modal characteristics analysis of a doubly clad optical fiber with semi-weakly guiding approximation. Opt. Commun..

[25] Cardoso E.S., Samad R.E., Motta C.C. Theoretical Investigation of Transverse Mode Instability in Yb-DCFs Due to Thermally-Induced Modal Coupling. Proceedings of the 2025 SBFoton International Optics and Photonics Conference (SBFoton IOPC).

[26] Monerie M. (1982). Propagation in doubly clad single-mode fibers. IEEE Trans. Microw. Theory Tech..

[27] Tsao C.Y.H., Payne D.N., Gambling W.A. (1989). Modal characteristics of three-layered optical fiber waveguides: A modified approach. J. Opt. Soc. Am. A.

[28] Erdogan T. (1997). Cladding-mode resonances in short- and long-period fiber grating filters. J. Opt. Soc. Am. A.

[29] Cardoso E.S., Samad R.E., Motta C.C. (2025). Theoretical analysis of transverse mode instability induced by the quantum defect in high-power YDCFLs. Proceedings of the 2025 SBMO/IEEE MTT-S International Microwave and Optoelectronics Conference (IMOC).

[30] Karimi M. (2018). Theoretical study of the thermal distribution in Yb-doped double-clad fiber laser by considering different heat sources. Prog. Electromagn. Res. C.

[31] Wisal K., Chen C.W., Cao H., Stone A.D. (2024). Theory of transverse mode instability in fiber amplifiers with multimode excitations. APL Photonics.

[32] Ward B., Robin C., Dajani I. (2012). Origin of thermal modal instabilities in large mode area fiber amplifiers. Opt. Express.

[33] Hansen K.R., Alkeskjold T.T., Broeng J., Lægsgaard J. (2012). Thermally induced mode coupling in rare-earth doped fiber amplifiers. Opt. Lett..

[34] Fan Y., He B., Zhou J., Zheng J., Liu H., Wei Y., Dong J., Lou Q. (2011). Thermal effects in kilowatt all-fiber MOPA. Opt. Express.

[35] Eilchi M., Parvin P. (2016). Heat generation and removal in fiber lasers. Fiber Laser.

[36] Tao R., Ma P., Wang X., Zhou P., Liu Z. (2015). Study of wavelength dependence of mode instability based on a semi-analytical model. IEEE J. Quantum Electron..

[37] Gloge D. (1972). Optical power flow in multimode fibers. Bell Syst. Tech. J..

[38] Zervas M.N. Transverse mode instability analysis in fiber amplifiers. Proceedings of the Fiber Lasers XIV: Technology and Systems.

[39] Zervas M.N. (2019). Transverse-modal-instability gain in high power fiber amplifiers: Effect of the perturbation relative phase. APL Photonics.

[40] Kelson I., Hardy A.A. (2002). Strongly pumped fiber lasers. IEEE J. Quantum Electron..

[41] Peysokhan M., Mobini E., Mafi A. (2020). Analytical formulation of a high-power Yb-doped double-cladding fiber laser. OSA Contin..

[42] Smith A.V., Smith J.J. (2012). Influence of pump and seed modulation on the mode instability thresholds of fiber amplifiers. Opt. Express.

[43] Dong L. (2022). Accurate modeling of transverse mode instability in fiber amplifiers. J. Light. Technol..

[44] Tao R., Ma P., Wang X., Zhou P., Liu Z. (2015). Influence of core NA on thermal-induced mode instabilities in high power fiber amplifiers. Laser Phys. Lett..

